# Influence of Wood Fly Ash on Concrete Properties through Filling Effect Mechanism

**DOI:** 10.3390/ma14237164

**Published:** 2021-11-24

**Authors:** Ivan Gabrijel, Marija Jelčić Rukavina, Nina Štirmer

**Affiliations:** Faculty of Civil Engineering, University of Zagreb, Fra Andrije Kačića Miošića 26, 10000 Zagreb, Croatia; marija.jelcic.rukavina@grad.unizg.hr (M.J.R.); nina.stirmer@grad.unizg.hr (N.Š.)

**Keywords:** biomass, wood fly ash, supplementary cementitious materials, filling mechanism

## Abstract

This paper presents the results of an experimental study aimed at determining the influence of wood fly ash (WFA) from three Croatian power plants on the properties of concrete. First, the chemical and physical properties of WFA’s were determined. It was found that these properties are highly influenced by combustion technology, the type and parts of wood used as fuel, and the local operating conditions. Subsequently, workability, heat of hydration, stiffness development, 28-day compressive strength, apparent porosity, and capillary absorption were determined on concrete mixes prepared with WFA as cement replacement from 5–45% by weight. Cement replacement up to 15% with the finest WFA accelerated hydration, stiffness development, and increased compressive strength of concrete up to 18%, while replacement with coarser WFA’s led to a decrease in compressive strength of up to 5% and had more gradual heat liberation. The dominant effect that could explain these findings is attributed to the filler and filling effect mechanisms. At the same time replacement content of up to 45% had very little effect on capillary absorption and could give concrete with sufficiently high compressive strength to be suitable for construction purposes.

## 1. Introduction

The need to reduce greenhouse gas emissions accelerates the transition to renewable energy sources. Biomass is the largest source of renewable energy in the European Union (EU), of which 60% comes directly or indirectly from forests [1,2]. The most common way of producing energy from biomass is combustion, and this process yields on average a quantity of ash between 2.7% and 3.5% of the original weight of wood biomass [3]. From the data on the quantity of wood used for energy production, it was estimated that about 7.3 million tons of ash were produced from wood biomass in the EU-28 countries in 2015 [4]. The primary energy supply of solid biomass during 2018 reached an amount of 47,260 PJ worldwide and 5704 PJ in the EU-28 [5]. Assuming that the combustion of 1 PJ of primary biomass produces about 2000 tons of ash [6], it follows that about 95 million tons of ash were produced globally and about 11 million tons of ash were produced in the EU-28 countries, most of which is wood biomass ash (WBA).

Despite the fact that different possibilities of WBA recycling have been recognized most of the ash is being landfilled [6,7,8,9,10]. Logistical problems, variations in the ash properties, contamination due to co-incineration with treated wood, lack of standards and legislation have been identified as obstacles in sustainable WBA management [11,12,13]. However, WBA disposed of in landfills can lead to air pollution with fine ash particles that can be carried by the wind, or they can contaminate soil or groundwater, so these landfills need to be properly designed and maintained [14,15,16]. In addition, the cost of landfilling is expected to increase, which will affect the price of heat and electrical energy [17,18]. Therefore, management of WBA in such a way that the balance is found between economic and environmental requirements is necessary.

Within the EU many biomass plants are burning wood residues to produce heat and/or electrical energy [17]. During combustion in a plant, two types of ash are generated: bottom ash and fly ash. Bottom ash is produced in the combustion chamber and comprises the coarse fraction which is formed by the total or partially burnt material, while fly ash is separated from the stream of gases outside the combustion chamber [11]. The ratio of bottom to fly ash varies depending on the combustion technology. In grate combustors, bottom ash usually accounts for 60% to 90% [6] while in bubbling fluidized bed combustors the bottom ash often represents 5% to 17% by weight [11]. Ash from the combustion of natural woody biomass contains valuable plant nutrients such as K, P, Mg, and Ca, most of which are contained in the bottom and coarse fly ash, while volatile heavy metals are concentrated in the fine fly ash fraction [6]. Therefore, it has been suggested that the bottom and coarse fly ash fraction should be returned to the forest from which they originated, while the fine fly ash should be utilized in industrial processes or disposed of [6]. 

The cement and concrete industry has been identified as one of the main potentials for biomass ash utilization [7]. The use of coal or biomass fly ash in concrete reduces the consumption of natural resources and reduces the CO_2_ emissions caused primarily through the reduction of Portland cement content in concrete [19]. Besides in the concrete industry, it has been shown that fly ash finds its application in various construction areas [20,21,22,23,24,25]. Pulverized coal fly ash has gained considerable importance as a supplementary cementitious material due to its pozzolanic properties, but also due to the improved workability of concrete attributed to the spherical shape and plain surface of ash particles. Both, the chemical composition and the shape of ash particles are influenced by the temperature of combustion [26,27]. In conventional pulverized coal-fired boilers, combustion takes place at temperatures between 1150 °C and 1750 °C which results in the melting of the majority of the minerals contained in the coal which is crucial for the formation of spherical particles [26]. If the temperature of combustion is below the melting point, such as in the case of fluidized bed combustion, coarse and irregular particles are formed [28]. The temperature of combustion of wood biomass usually takes place at temperatures up to 1000 °C which is below the melting point for most species present in the biomass [29]. Using a scanning electron microscope, it was found that wood fly ash (WFA) particles range from spherically fused to irregularly shaped, and porous particles, particles that look like conglomerates of smaller particles and particles with regular smooth edges [4,30]. The chemical composition of WFA is different from that of the coal fly ash. It usually contains more alkali and less alumina than coal fly ash. Besides, the chemical compositions of WFA vary more than that of coal fly ash because it depends on the wood species, parts of wood being used as a fuel, and the season of biomass harvest [17,31].

The application of WFA is currently outside the scope of the standard for fly ash for concrete (EN 450-1:2012) [32]. Extensive research has been conducted to investigate the possibilities of using WFA as a cement replacement material [30,31,33,34,35,36,37,38,39,40,41]. It has been reported that WFA from grate combustors and fluidized bed combustors can have hydraulic and/or pozzolanic properties [30,34,37]. The utilization of WFA as a cement replacement modifies the workability and mechanical properties of the tested material. As the cement replacement level increases, water demand usually increases and the compressive strength decreases [30,31,33,34,35,36,38]. However, replacing cement with WFA can also increase compressive strength when the cement replacement level is low [35,42]. Improved workability has also been found when WFA was used as a filler and partial replacement for fine aggregate in concrete [37].

Most of the studies on the influence of WFA produced during combustion in biomass plants on the properties of cement composites have been tested on cement pastes and mortars and only a small number of experiments have been scaled up to the concrete level. To develop guidelines for the use of WFA in structural concrete, the interdependence between properties of WFA and the properties of fresh and hardened concrete must be clearly established. 

The aim of the experimental work presented here is to show the influence of WFA with different physical and chemical properties, used as partial cement replacements, on the properties of fresh and hardened concrete and to identify the most probable mechanisms that govern these changes. The WFA used originates from three different power plants with two types of incineration technologies.

## 2. Materials and Methods

### 2.1. Characterisation of Fly Ash and Cement

Wood fly ash (WFA) is collected from three powerplants in Croatia. All three plants are co-generation biomass plants producing both heat and electrical energy. Plant F4 is the smallest plant with 1 MW of electrical capacity and 4.1 MW of heat capacity. It is located in the mountain part of Lika-Senj County. Wood species mostly used as a fuel are given in Table 1 and the parts of wood used as a fuel consist of wood chips made from roundwood and thinning residues, including twigs, tops, and branches. Plant F5 operates in the northern part of Croatia in Varazdin County, and it has a production capacity of 2.75 MW of electrical and 15 MW of heat energy. It uses wood chips made from roundwood and thinning residues, including twigs and tops, branches, bark, needles/leaves, and wood industry waste (including bark). Plants F4 and F5 use fixed bed combustion in which wood fuel is burned in a grate furnace [43]. During the combustion process, fly ash F4 and F5 are collected by cyclones [43]. Plant F6 is the largest biomass plant with the capacity of production of 8.6 MW of electrical and 16 MW of heat energy and is in the eastern part of Croatia in Vukovar-Srijem County. Wood fuel is used in the form of wood chips made from whole trees containing bark, twigs, and leaves. Plant F6 uses a bubbling fluidized bed combustion system with quartz sand as s bed material [43]. The fly ash particles carried from the combustor are captured by bag filters [43]. Fly ash captured by bag filters is finer than fly ash captured by cyclones [30].

The information about the incineration temperature of wood biomass is given by the technologists in the power plant. The chemical and physical properties of WFA used in this work are presented in Table 1. The designations for wood ash used in this paper correspond to the F4, F5, and F6 in a paper [35] where the same ash was used to study the effect of cement replacement on the properties of cement paste and mortar. In the paper [35] additional information is given on the chemical and physical properties of WFA. 

**Figure 1:**
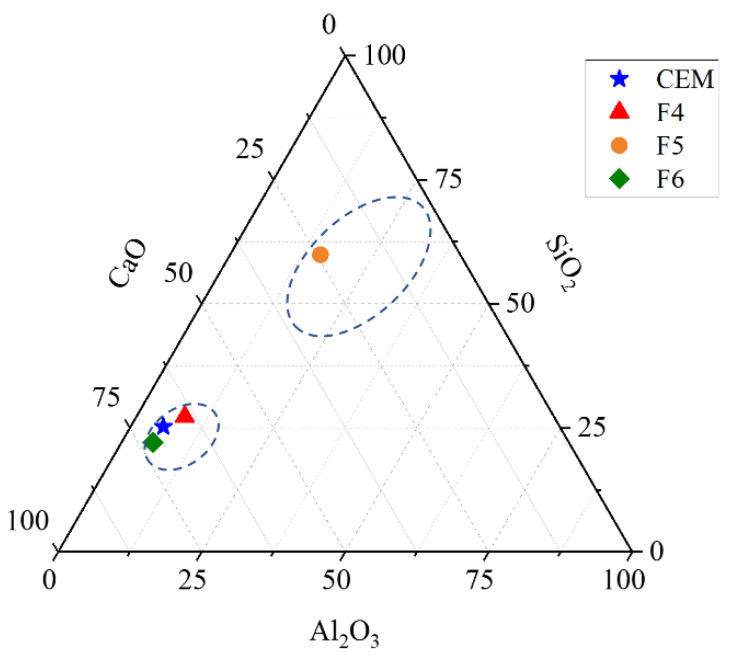
Ternary plot CaO-Al_2_O_3_-SiO_2_ for the wood fly ash used in the experimental work. The area on the graph characteristic for Portland cement and coal fly ash is taken from [44].

The ternary diagram in Figure 1 shows that WFAs F4 and F6 have CaO-Al_2_O_3_-SiO_2_ ratios similar to Portland cement, while ash F5 has an oxide ratio in the area of coal fly ash [44]. WFA F5 also has the highest content of pozzolanic oxides, which is higher than the minimum pozzolanic oxides for class C pozzolans according to ASTM C618-19 standard [45]. 

The particle-size distribution (PSD) was analyzed by a laser diffraction method using a dry measurement (Shimadzu SALD 3101 Instrument, Kyoto, Japan). Elemental composition is determined by X-ray fluorescence according to standard ISO/TS 16996:2015 [46]. The amount of CaCO_3_ is determined by thermogravimetric analysis (TGA) (for details see [4]). Loss on ignition (LOI) has been determined according to ASTM D7348-13, density according to ASTM C188-17, and pH value according to EN 12176:2005 [47,48,49]. The morphology of the WBA samples was analyzed by scanning electron microscopy (SEM) using the instrument SEM FE MIRA II LMU. Prior to imaging a suitable amount of powder is placed on a conductive adhesive tape and sputtered with gold/palladium in argon plasma. SEM micrographs were obtained in high vacuum mode under pressure of (3–5) ×10^−4^ Pa and 10 kV settings.

**Figure 2:**
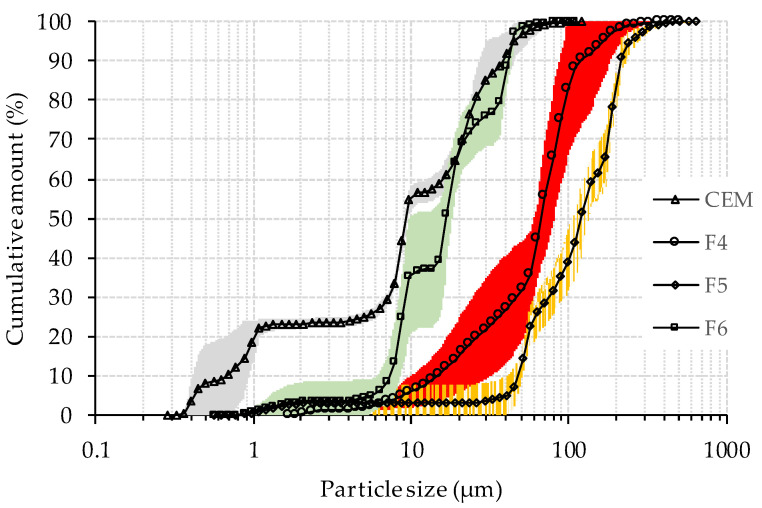
Particle size distribution of cement and WFA (shaded area represents standard deviation).

The PSD for cement and WFA is presented in Figure 2 and the median particle size (*d*_50_) is given in Table 1. Fly ashes F4 and F5 produced by grate combustion technology contain coarser particles than ash F6 produced by fluidized bed combustion which is in agreement with literature data [30]. The specific surface area (SSA) of cement and WFA is calculated from the PSD assuming spherical particles (Table 1). The SSA of cement particles in Table 1 is 1.3, 3.6, and 4.4 times larger than the specific surface area of wood fly ash F6, F4, and F5, respectively.

### 2.2. Concrete Mix Design

WFA was stored in the laboratory for a period of approximately six months before being used in concrete. During storage, WFA was kept sealed in plastic bags and then stored in closed plastic containers.

**Figure 3:**
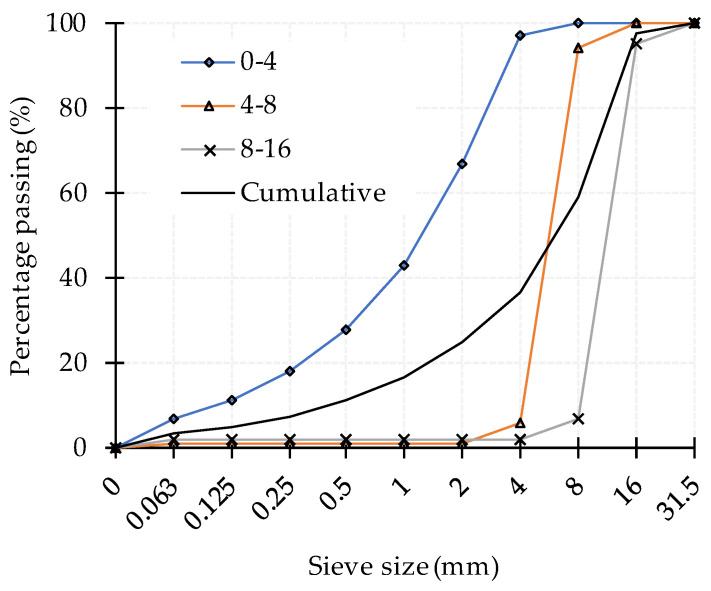
Aggregate grading curves.

The cement used in this study was Portland cement type CEM I 42.5 R conforming to European standard EN 197-1:2011 [50]. The aggregate was crushed dolomite with an average bulk density of 2.8 kg/dm^3^ and absorption of 0.47%, 0.66%, and 0.32% for fractions 0/4, 4/8, and 8/16 mm, respectively. The particle size distribution curves are presented in Figure 3.

The compositions of the concrete mixes are given in Table 2. Each mix is designated according to the WFA used and the cement replacement percentage. All mixes had the same water/(cement + WFA) ratio of 0.5. A total of 7 concrete mixes containing WFA were prepared with cement replacement percentages of 15% and 30% for ash F4, 15%, 30% and 45% for ash F5, and 5% and 15% for ash F6. In the first stage of the experimental work, a reference concrete mix (M0) and mixes with 15% cement replacement were made. The decision to increase or decrease the cement replacement level in the further mix design process was based on the influence of WFA on the workability of the fresh concrete. Workability was evaluated by the slump test, and cohesiveness was determined by visual inspection of each mix.

The mixing of the concrete was performed in a compulsory mixer with a capacity of 75 L. Before mixing, all constituents were conditioned to a temperature of 20 ± 2 °C. The mixing procedure described in the standard EN 480-1:2014 [51] was used for the preparation of the reference mix. Following this procedure, aggregates and approximately half of the mixing water were added to the pan and mixed for 2 min, and then mixing was stopped for 2 min. At the end of this period, cement and WFA were added to the mixer. The components were mixed for 30 s and during the next 30 s the remaining water was added. Mixing was then continued for an additional 2 min. The entire mixing process lasted 7 min.

### 2.3. Testing Methods

The following properties of concrete were measured in the fresh state: consistency by slump test (EN 12350-2:2019), density (EN 12350-6:2019), air content (EN 12350-7:2019), and temperature [52,53,54]. The concrete was compacted on a vibrating table. After compaction, the specimens were stored in a room with a temperature of 20 ± 5 °C and covered with a plastic sheet. After 24 h, the specimens were demoulded and moved to a curing room where they were cured in the air at a temperature of 20 ± 2 °C and a relative humidity of >95%. All measurements were performed on concrete from the same batch.

The heat of hydration is monitored with the ToniCAL 7336 heat flow differential calorimeter. During the measurement, the calorimeter was placed in the room where the temperature was maintained at 19 ± 2 °C. The measuring vessel of the calorimeter contains two measuring cells, one for the concrete specimen and another for the inert (reference) specimen. The inert specimen was maintained at the preselected temperature of 23 °C. All concrete components were tempered prior to mixing so that the temperature of the fresh concrete was as close as possible to the temperature of the inert specimen. After mixing the fresh concrete, a specimen of concrete was compacted in a cylindrical steel mould with a diameter of 150 mm and a height of 300 mm, and the mould was then placed in the calorimeter vessel. The heat generated by the hydration of the cement was evaluated from the voltage difference detected by the thermoelectric conductors placed around the specimens. The rate of heat generation was monitored during the first 5 days of hydration. During the heat generation measurement, the temperature of the concrete specimen changes and it is measured by a Pt-100 probe approximately in the center of the concrete specimen. The initial temperature of the specimen was between 22–23 °C, it increased depending on the rate of heat generation in the specimen, reached a maximum, and then decreased to the preselected temperature of 23 °C. The measurement of the temperature is complementary to the measurement of the heat flow and provides important information for the interpretation of the results, because the increase of the temperature additionally accelerates the hydration, so the heat evolution occurs at a higher rate than would be the case under isothermal conditions, but also at a slower rate than in the semi-adiabatic calorimeter. 

Concrete stiffness development was monitored by measuring ultrasonic pulse velocity (UPV) at 1, 2, 7, and 28 days of age using a portable ultrasonic instrument with 54 kHz longitudinal wave transducers. The measurement was performed on concrete cubes with a side length of 150 mm. The same cubes were used for testing the compressive strength of concrete at 28 days of age according to standardized procedure (EN 12390-3:2019) [55].

The capillary absorption measurement was performed on cylinder specimens 150 mm in diameter and height 50 mm, obtained by sawing from the standard cylinder 150 mm in diameter and height 300 mm. Upper and bottom slices of the cylinder were not tested to avoid effects of different boundary conditions. The first 10 mm of side surface in contact with water was coated with epoxy resin. Prior to testing, the specimens were oven-dried at a temperature of 105 ± 5 °C until the change in mass for two consecutive weighing became less than 0.5 g (≈0.025 % of mass). After cooling to ambient temperature, the specimens were placed in a water container on cylindrical rods, and the water level was adjusted so that the bottom surface of the specimens was immersed in the water 2–5 mm. The mass of the specimens was measured at an interval of 5, 15, 30, 60, 120, 240, and 1500 min. From each mix, three specimens were tested for capillary absorption.

After the capillary absorption test, the water level in a water container was gradually increased at the rate of approximately ¼ of the height of the specimens per day until the specimens were completely immersed. The specimens were kept under water until the mass of the specimens changed by less than 0.5 g after two consecutive measurements. The specimens were then weighted in surface dry conditions in air and in water. Bulk density in dry (*ρ_z,dry_*) and saturated conditions (*ρ_z,sat_*), apparent solid density (*ρ_a_*), and apparent porosity (*p_a_*) were calculated according to Equations (1)–(4). The term apparent is used here because it is assumed that only open pores are filled with water.

(1) $$\rho_{z,dry}=\frac{m_{dry}\rho_{w}}{m_{sat}-m_{sat,w}},$$

(2) $$\rho_{z,sat}=\frac{m_{sat}\rho_{w}}{m_{sat}-m_{sat,w}},$$

(3) $$\rho_{a}=\frac{m_{dry}\rho_{w}}{m_{dry}-m_{sat,w}},$$

(4) $$p_{a}=\frac{m_{sat}-m_{dry}}{m_{sat}-m_{sat,w}}\cdot 100,$$

where *m_dry_* is the mass of dry material, *m_sat_* is the mass of saturated material, *m_sat,w_* is the mass of saturated material weighted in water, and *ρ_w_* is the density of water (1000 kg/m^3^).

## 3. Results and Discussion

### 3.1. Fresh Concrete Properties

The density of the fresh concrete was determined using a measuring container of 8 liter capacity. The density varied between mixes in the range of 2440–2500 kg/m^3^. For ashes F4 and F5, the density slightly decreased with increasing WFA content, while for ash F6, the density increased with increasing ash content. This observation on the fresh concrete was confirmed by the results of the bulk density measurements on the hardened concrete (Table 3). 

The air content was low in all mixtures, indicating dense packing of the concrete constituents after compaction on the vibrating table. The highest air content in mix F6-15 may be partially attributed to the low slump value, which made the compaction of concrete harder.

The initial temperature of the concrete varied between 22.2 and 25.1 °C. Only mixes with ash F4 showed a consistent increase in temperature with the increase in ash content. This could be caused by fast initial heat liberation on contact with water but could also be attributed to temperature variations of the concrete constituents prior to mixing.

Replacing cement with WFA changed the workability of concrete. It is evident that both the properties of the ash and the replacement level had a large influence on cohesiveness and fluidity. It is important to emphasize that all the mixes had adequate workability to be placed in moulds and compacted on a vibrating table without the loss of homogeneity.

The influence of cement replacement on workability, as determined by the slump test, is shown in Table 3. Ash F6 at 15% cement replacement had the largest influence on slump values, where slump decreased from 90 mm measured on the reference concrete to 5 mm. Concrete produced with 15% cement replacement with F4 and F5 ashes showed only minor deviations from the slump value measured on the reference mix. A significant decrease in slump was also present with the F4-30 mix. The cement mix with ash F5 had almost no influence on the slump values. However, mix F5-45 gave a harsh concrete typical for low cement content concrete [56]. This mix also showed increased bleeding and a tendency for the segregation of the largest aggregate particles. It is also interesting to note that the F4-15 and F6-5 mixes showed an increased slump compared to the reference mix. This increase in slump is within the reproducibility limits for the test method (EN 12350-2:2019) [52] but may also be a reflection of the net effect caused by the cement replacement with WFA, as discussed hereafter.

No clear conclusion can be drawn from the previously published data on the impact of WFA on the water demand of concrete mixes, as both increased and decreased water demand have been reported. Increased water demand or decreased workability in mixes with WFA was attributed to the irregular shape and higher specific surface area due to the porous WFA particles [31,57,58]. Berra et al. [37] tested two types of WFA at cement replacement levels of 15% and 30% and found that the workability of hardened cement paste improved with the increasing replacement of cement by WFA for ash with finer particles (*d*_50_ < 60 µm), while the opposite was found for ash with coarser particles (*d*_50_ ≈ 100 µm) [37]. It was suggested that the improved workability of mixes containing WFA was due to the lower ash dissolution and lower loss on ignition of the finer ash. The same authors also found that the addition of fine WFA instead of limestone filler improved the workability of concrete. 

Changes in the workability of concrete caused by mineral admixtures are attributed to the various mechanisms of interaction between solid particles. Many of these mechanisms are governed by the physical properties of the solid particles such as fineness, shape, surface texture, and porosity of the particles, but they also depend on grain composition, mix proportions, and the presence of other admixtures [56,59,60,61]. Several hypotheses have been proposed to explain the improved workability of mixtures containing mineral admixtures:

- Reduced interparticle friction during flow caused by the spherical shape and plain surface of admixture particles—ball-bearing effect [60].
- Fine particles fill the void space between relatively large cement grains and release entrapped water and enhance fluidity—filling effect [60]. The circularity of the particles goes in favor to this mechanism [62].
- Adsorption of admixture particles on the surface of the cement particles due to electrical charges, which deflocculates the cement particles and increases mobility [59].

On the other hand, the reduction of workability due to the addition of mineral admixtures is often attributed to:

- Increase in solid surface area due to the presence of very fine particles that have a tendency to adsorb water [56,59].
- Replacement with a filler containing large particles (>45 µm) [60].
- Open porosity of the particles which increases the specific surfaces [59,61].

**Figure 4:**
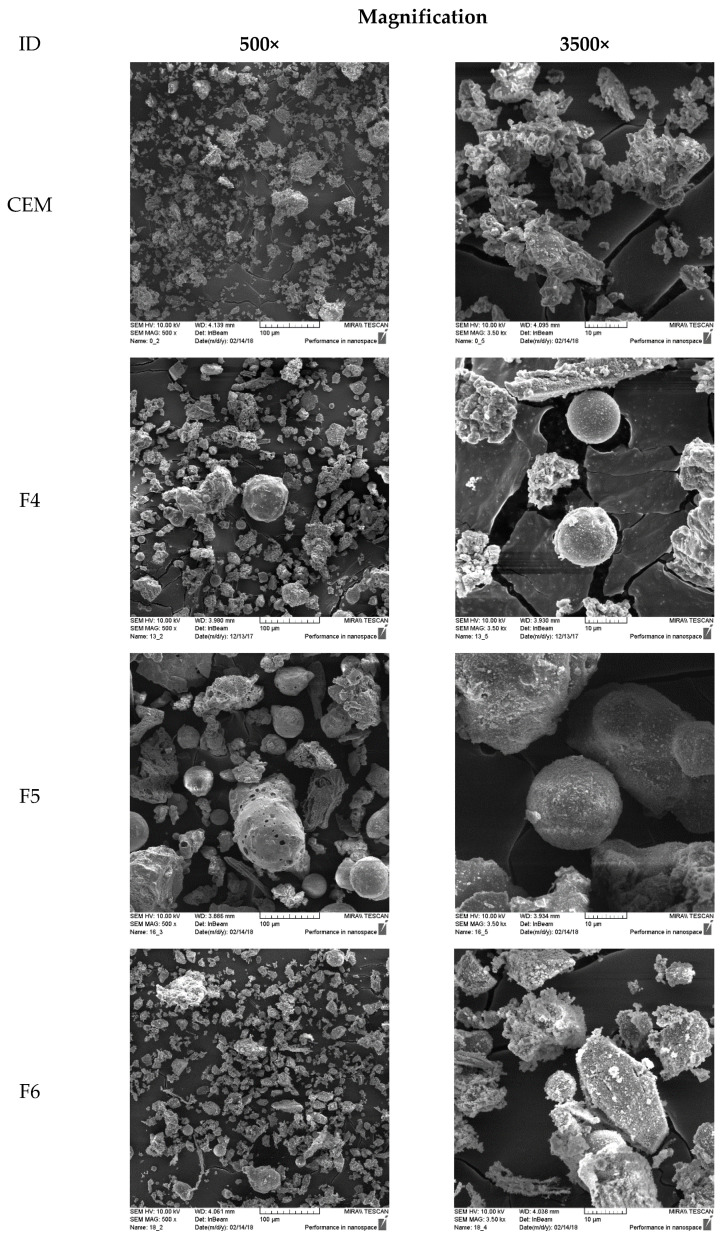
SEM images at 500× and 3500× magnification of cement and WFA.

Considering the SSA from Table 1, the replacement of cement with WFA resulted in a reduction in the surface area of the solid phases in the concrete. However, the assumption of spherical particles probably underestimates the actual surface area [63]. For typical cement powders, the SSA calculated for spherical particles should be multiplied by a factor of 1.6–1.8 [64]. The fluidized bed combustion technology in coal-burning processes produces sub-angular particles resulting in a surface area up to 5 times larger compared to pulverized coal fly ash with typically spherical particles, due to lower combustion temperature [26]. This contributes to its reactivity and increases water demand [65]. SEM images show that all the WFAs used in this work contain both irregular and spherical particles and the main difference is in the size of the particles (Figure 4). Compared to the WFA particles, the cement particles seem to be more irregular, which is to be expected since these particles were formed by crushing larger clinker grains. Therefore, replacing cement with WFA increases the “sphericity” of the particles. Another important effect on workability is the water absorbed by the porous particles. The main content of the LOI in fly ash is unburned carbon, which has a high porosity and a very large specific surface and can absorb a significant amount of water [61]. The above-mentioned effects interfere and create the overall effect on concrete workability. Ash F6 contained particle sizes in a range very similar to Portland cement. The addition of ash F6 to the concrete increases the sphericity of the particles, which, in combination with the small particle sizes improved the packing and enhanced the workability.

It is known that the addition of fine mineral admixtures enhances cohesiveness and reduces the size and volume of voids [56]. The reduction of voids, i.e., improved packing in specimens from mixes containing ash F6, is confirmed by the increased density, reduced porosity, and increased compressive strength (Table 3). Ash F6 also contained the highest amount of unburned carbon (LOI 12.7%), which absorbed a certain quantity of water. The increase in slump in mix F6-5 could be attributed to the enhanced workability due to the filling effect, while at 15% replacement the effect of water absorption and enhanced cohesiveness become dominant. The F4 ash contained coarser particles than the Portland cement, but it also contained 30% of particles <45 µm. This quantity of fine particles together with the increased circularity of the particles enhanced fluidity. At the same time, the portion of particles >45 µm loosens the particle packing, leading to increased water demand. The increase in slump in mix F4-15 could be attributed to the reduced water demand due to the filling effect, while at 30% cement replacement, the loosening of particle packing becomes dominant. Ash F5 contains only 7% of particles <45 µm, so it does not have capability to reduce the water demand through the filling effect. A loose particle packing is created, which decreased the cohesiveness of the mix, while part of the water was probably absorbed by the unburned carbon particles (LOI 8.3%).

**Figure 5:**
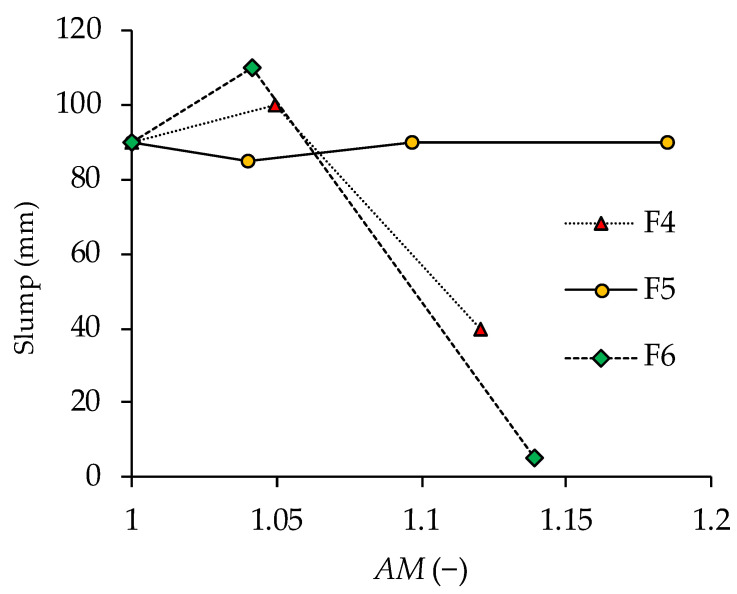
Slump vs. area multiplier (*AM*) in concrete.

Oey et al. [63] characterize the influence of cement replacement by powder addition with an area multiplier *AM*, which evaluates the increase in solid surface induced by filler addition:

(5) $$AM=1+\frac{r_{}SSA_{filler}}{(100-r)SSA_{cement}},$$

where *r* represents the mass percentage replacement of cement by filler. A larger value of *AM* indicates that the filler is finer or that it is present in a larger amount. According to the *AM* value, the 5% replacement with ash F6 is approximately equal to the 15% replacement with ash F4 or F5. The 15% replacement with ash F6 gives a larger *AM* value than the mix with 30% replacement of ash F4 and F5. In Figure 5 slump results are plotted against the *AM* value. The correlation between slump and *AM* in mixtures containing ash F4 and F6 shows a similar trend, indicating that there is a cement replacement level where workability is increased by WFA addition. Mixes containing ash F5 do not show the same correlation between slump and *AM* value, as mixes made with ash F4 and F6, which is attributed to the particle size distribution of ash F5.

The existence of optimum WFA content in terms of concrete workability has been observed by Yang et al. [57] and attributed to improved packing of particles when part of the sand was replaced by ash. Increasing the sand replacement level above the optimum value leads to a decrease in slump compared to the mix without ash [57]. Skripkiunas et al. [66] tested the rheological properties of cement pastes made with 0, 5, 10, 20, and 30% cement replaced with biomass fly ash. They found that only cement paste made with 10% biomass fly ash had lower yield stress and lower viscosity than a mix without fly ash. Although the authors did not discuss the reasons for their findings, it is interesting to note that the Portland cement and biomass fly ash used in [66] had a median particle size of 9.94 µm and 23.97 µm, which is similar to a median particle size of the Portland cement and ash F6 used in this work (Table 1).

All three ashes used in this work had a large amount of K_2_O content (Table 1), which can increase the reactivity of the C_3_A present in the cement and additionally increase the water demand [67]. Increasing the cement replacement also increases the content of alkalis in the mixture. In Portland cement, the ettringite formation from C_3_A and its precipitation on the surface of the cement particles or in the pore solution is the main cause of the gradual loss of workability in cement pastes [68,69]. It was shown that ettringite is the main product during self-hardening of wood fly ash from fluidized bed combustion having high Ca content, while gypsum is formed as the main product in ashes with low Ca content [70,71]. Increased reactivity of C_3_A caused by increased alkali content would also contribute to early heat generation, resulting in increased temperature of fresh concrete [34,72].

### 3.2. Heat Generation

**Figure 6:**
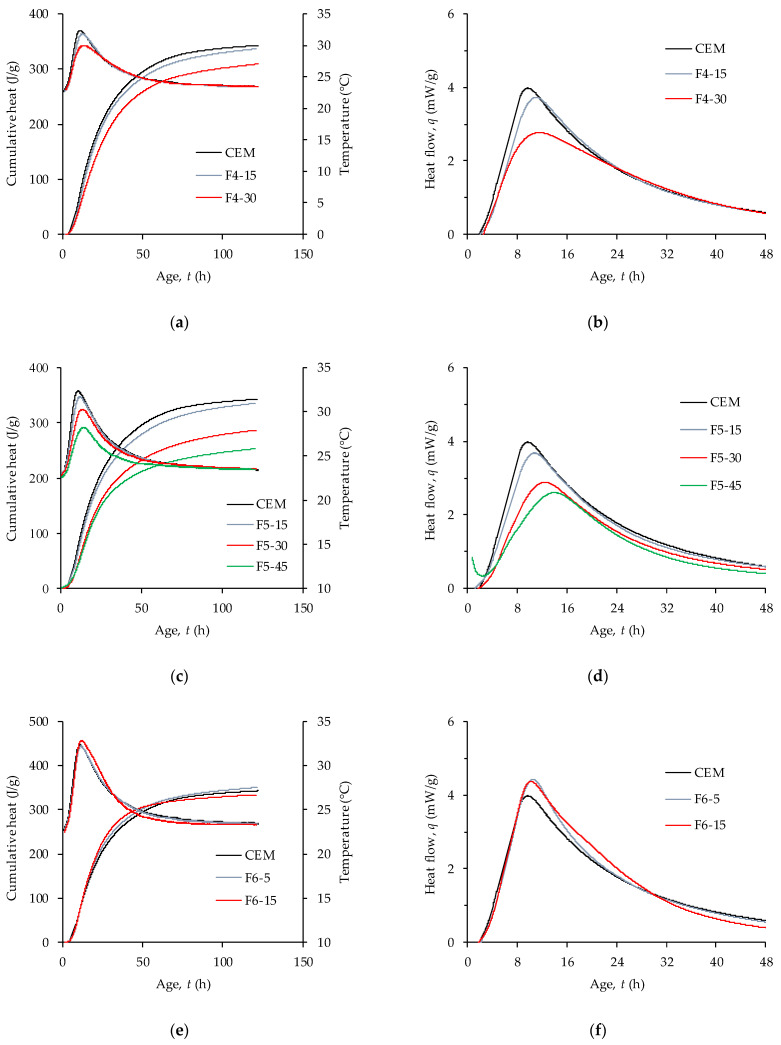
Temperature, heat flow, and cumulative heat measured on concrete specimens: (**a**,**b**) comparison between reference mix and mixes containing WFA F4; (**c**,**d**) comparison between reference mix and mixes containing WFA F5; (**e**,**f**) comparison between reference mix and mixes containing WFA F6.

Measurements of heat output started 30–90 min after initial contact of cement and water due to the time needed to perform tests on fresh concrete and, if necessary, the additional time required to precondition the specimen so that the concrete temperature is as close as possible to 23 °C. The temperatures measured in the hydrating specimens are plotted in Figure 6. The difference between initial and maximum temperatures has been within 5–10 °C, with the lowest value found in mixture F5-45 and the highest value in mixture F6-15. The temperatures measured in the specimens are also representative of the heat flow from the specimen. 

The results of heat generation measured on concrete mixtures containing WFA are presented in Figure 6. The heat output is related to the mass of the whole system cement + WFA. Replacing 15% of the cement with WFA resulted in almost the same amount of heat liberated during the first 5 days for all 3 ash types (336 J/g, 335 J/g, and 334 J/g for F4, F5, and F6, respectively). Compared to the heat liberated from the mixture without WFA, this is a reduction of 2–3%. In the mixture containing 5% replacement of F6 ash, the total heat liberated after five days increased by 2% compared to the mixture without WFA. Increasing the WFA content to 30% further reduced the amount of heat liberated by 10% and 17% for mixtures with F4 and F5 ash, respectively. Replacing 45% of the cement with ash F5 reduced the amount of heat liberated by 26% over five days. Less emitted heat in calorimetric measurements can be explained by the fact that WFA has no binding or pozzolanic properties [42]. 

All the heat flow curves in Figure 6 have one significant peak separating the acceleration and deceleration periods of heat release. Replacement of cement with F4 and F5 ashes decreased the peak value of heat flow. Furthermore, the higher the ash content, the larger reduction of heat generation is. At a 15% replacement level, these ashes had almost the same effect on the heat flow. The difference in heat flow between the mixtures with F4 and F5 ashes appeared at a 30% replacement level, and it can be seen that in the mixtures made with F5 ash, there is a larger delay in the peak heat flow and the slope of the curve decreases. Increasing the replacement level to 45% further delayed the hydration process. Effects on heat flow similar to those of ash F5 have been reported for mixtures of Portland cement and type C coal fly ash and have been attributed to the disruption of the aluminate-sulfate balance in mixtures containing >20% fly ash [73]. Roszczynialski and Nocun-Wczelik [74] also reported that low SO_3_ content relative to aluminate content produced a significant peak after 13 h caused by aluminate hydrates. Ash F5 has a significantly higher ratio of Al_2_O_3_ to SO_3_ than cement or both ash F4 and F6 (Table 1), so this could be the main cause of the changes in the course of early hydration in mixtures containing ash F5.

Contrary to mixtures containing ash F4 and F5, mixtures containing ash F6 in both replacement levels had an increased rate of heat liberation compared to the reference mixture. It is well known that even inert mineral admixtures, when blended with cement, can accelerate hydration [44,63,75]. Two mechanisms are often used to explain this effect, which is usually referred to as the filler effect. When mineral admixture contains very fine particles, extra surface area becomes available for nucleation of hydration products, resulting in a reduced thickness of impermeable C-S-H on C_3_S grains [67]. The second mechanism is related to the increased *w/c* ratio caused by the replacement of the cement. Increasing the *w/c* ratio decreases the early kinetics, but the long-term degree of hydration is increased as more water is available for hydration and at the same time there is more space for hydration products [76]. Besides these two effects, additional water becomes available for hydration due to the filling effect already explained in the previous section. The increased cohesiveness of the fresh concrete, the reduced porosity (Table 3), and the increased hydration rates found in mixtures with ash F6 (Figure 6e,f) can all be related to the filler effect. The replacement of cement with ash F6 probably activates all three of the above-mentioned mechanisms, while the effect of the filler in mixtures containing ash F4 and F5 is limited to the effects of increased *w/c* ratio, due to their coarse particles.

Accelerated hydration which manifested through increased maximum rate of hydration or a shift of the hydration rate curve toward an earlier age, has been found in several papers dealing with biomass ash [34,42,77]. Rissanen et al. [34] tested fluidized bed combustion of WFA milled to a PSD close to that of cement and found that at 10% cement replacement, the heat of hydration was higher than in the reference mortar mixture. Accelerated hydration of calcium hydrates in the presence of fly ash has been reported for cement paste with milled WFA, where 70% of the particles passed through 63µm sieve [42]. The shift in the hydration rate curve for cement pastes with fly ash from the burning of wheat and rice straws has been attributed to the high surface area of the specimens, that can act as nucleation sites [77].

### 3.3. Compressive Strength

Compressive strength was determined on four specimens from each mix. The average 28-day compressive strength and standard deviation are given in Table 3. For both ash F4 and F5, the compressive strength decreased with the increase of ash content in the concrete but for the mixtures with ash F6, the compressive strength increased with the increase of ash content. As already mentioned in Section 3.1, the replacement of cement with ash F6 resulted in the densest particle packing which could be the main reason for the increase in compressive strength.

**Figure 7:**
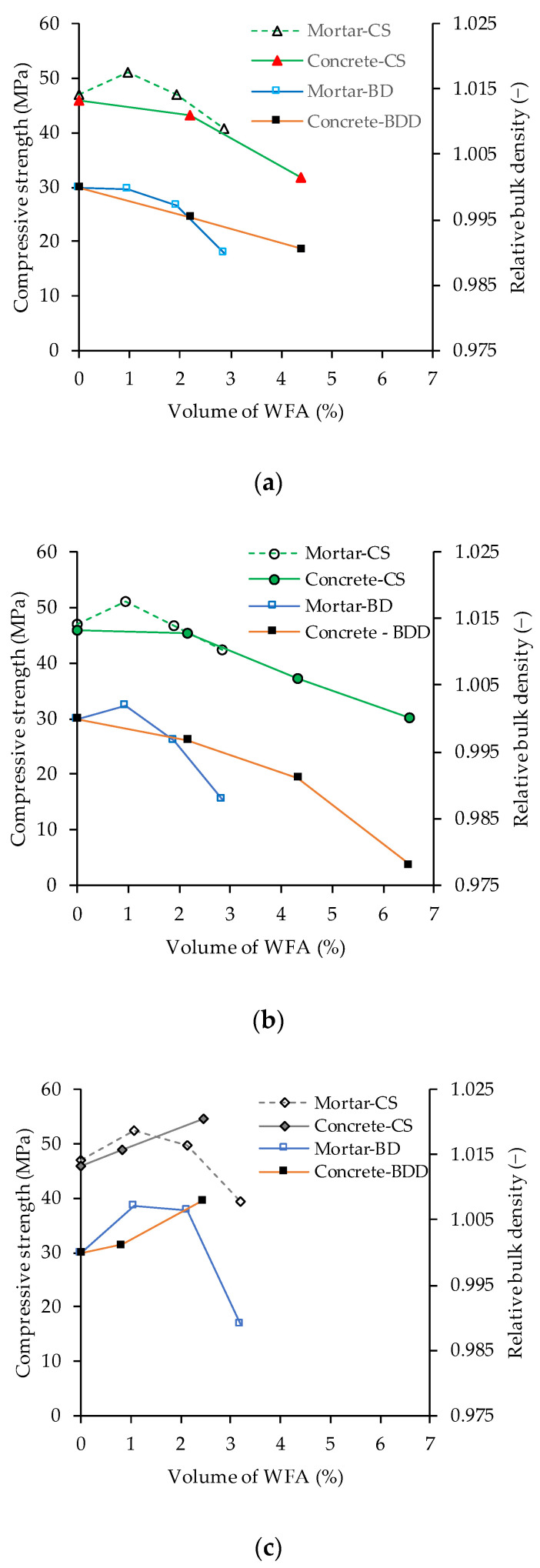
Compressive strength and relative bulk density of concrete and mortar mixtures with different volumes of WFA: (**a**) ash F4, (**b**) ash F5; (**c**) ash F6 (CS—compressive strength, BD—bulk density, BDD—bulk dry density).

Figure 7 compares the average 28-day compressive strength of concrete with the compressive strength of mortars made with 5%, 10%, and 15% cement replaced with ashes F4, F5, and F6 (results published in [35]). All mortar mixtures had the largest 28-day compressive strength at 5% replacement of cement with WFA. Further increase in ash content resulted in a decrease in compressive strength. The compressive strength data in Figure 7 are plotted against the volume of WFA in the mixture instead of the percentage of cement replacement because mortar contains a larger volume of WFA than concrete for the same cement replacement due to the larger cement to aggregate ratio. The results presented in Figure 7 show that there is a good agreement between the compressive strength determined on mortar and concrete. The results also indicate that the compressive strength of concrete made with F4 and F5 ashes could be improved at a cement replacement level of less than 15%.

In Figure 7, the average bulk density is given along with the compressive strength data and is expressed relative to the bulk density measured on the mixture without WFA. The bulk density of the mortar is determined from the mass and dimensions of the specimens before the compressive strength test. The results show that the increase in bulk density is followed by an increase in compressive strength, indicating that the main contribution to the increase in strength is related to the denser particle packing. An increase in compressive strength in mixtures made with WFA has been achieved by additional milling of the ash, which resulted in a better particle size distribution [42,78].

The reduction in compressive strength in WFA concrete may also be related to decreased aggregate content and the simultaneous incorporation of ash particles with lower stiffness and strength into the cement matrix. The density of WFA is lower than the density of cement, so the same mass of WFA occupies a larger volume than cement. This results in a reduction in the volume of aggregates in mixtures containing WFA (see Table 2). Since aggregates have greater strength and stiffness than WFA, their replacement by WFA weakens the concrete structure. Grau et al. [79] analyzed the compressibility and shear modulus of wood ash and mixtures of standardized quartz sand and wood ash. They found that wood ash had higher compressibility than sand and consequently its incorporation into sand/ash mixtures increased the compressibility of the mixtures Yang et al. [57] attributed the decrease in compressive strength of concrete containing wood ash, to the incorporation of weak, unburned carbon particles into the cement matrix. Several investigations showed that an increase in the WFA content in concrete was accompanied by a decrease in strength [9,31,42,58,72,80].

The decrease in strength may also be related to the lower content of cementitious material when part of the cement is replaced by WFA, as its reactivity is lower than that of the cement. It has been observed that WFA from fluidized bed technology hardens when mixed with water and reaches a 28-day compressive strength of up to 6 MPa [70,71]. A significant increase in compressive strength between 28 days and 1 year indicates a slow reaction [71]. Analysis of WFA-water mixtures showed that the main hydration products of fluidized bed fly ash are ettringite and various Ca-Al phases [71]. By using XRD analysis of 7 and 28 days aged hardened cement paste, it was found that the replacement of cement by WFA did not radically change the hydration products and that the changes were primarily related to the species present in the WFA [34]. The contribution of pozzolanic reactions to strength becomes significant after 7 or 28 days of hydration [81]. Therefore, the hydration products of WFA are expected to be very limited in the period up to the 28-days.

Estimation of the potential reactivity of WFA is usually based on its chemical composition. Berra et al. [37] assumed that the hydraulic index, defined as (CaO + MgO + Al_2_O_3_)/SiO_2_, could serve as a good indicator of hydraulic activity when its value is >1. Rajamma et al. [72] expected that WFA that contained >25% of CaO would be able to react hydraulically. Sigvardsen et al. [82] expect that there would be little or no pozzolanic activity when the amount of primary oxides (SiO_2_ + Al_2_O_3_ + Fe_2_O_3_) is <25%. However, the sum of the pozzolanic oxides does not have to be directly proportional to their pozzolanic activity [31,37,42]. It was pointed out that selective dissolution is a good method for estimation of hardening properties of ash from fluidized bed combustion processes [71].

The hydraulic index of ashes F4, F5, and F6 from this study are 3.1, 0.8, and 3.9, respectively, which according to [37] indicates that ashes F4 and F6 can contribute to strength mainly through hydraulic reaction. Fly ash F5 had the quantity of pozzolanic oxides > 50% (Table 1) and its CaO-SiO_2_-Al_2_O_3_ ratio is close to that of coal fly ash (Figure 1), therefore it had the highest potential for pozzolanic reactivity. Compressive strength tests on mortars up to 90 days of age containing ash F5 indicate that its pozzolanic activity is low probably due to its coarse particles [35]. Less emitted heat in calorimetric measurements can be explained by the fact that WFA does not have binding or pozzolanic properties [42]. The results of the calorimetric measurements (Figure 6) indicate that the F4 and F5 ashes have very low contribution to the strength development through chemical reactions during the first days of hydration. 

### 3.4. Stiffness Development

**Figure 8:**
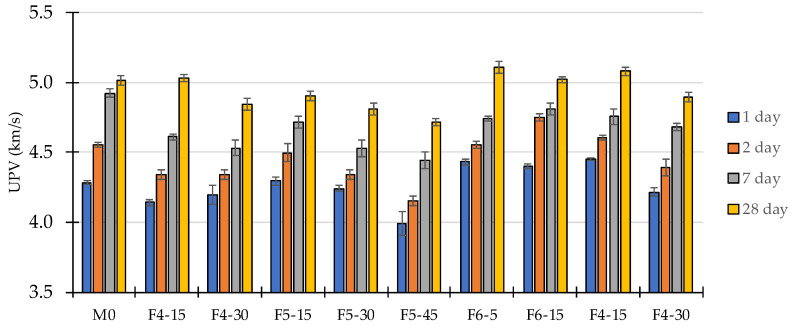
UPV measured on concrete specimens at 1, 2, 7 and 28 days of age.

The increase in UPV of longitudinal waves in early-aged concrete is related to the increase in Youngs and shear modulus [83,84]. A comparison of UPV values measured at four different ages is presented in Figure 8. The lowest UPV values have been measured on specimens made from concrete containing the largest volume of WFA. Compared to the UPV values measured on the reference mixture, the average decrease was 8% (F5-45) and 4% (mixtures with 30% cement replacement). These lower UPVs can be related to the increased compressibility caused by the incorporation of particles with lower stiffness into the cement matrix. In mixtures containing up to 15% WFA, the 28-day UPV value is within ±2% of the value measured on the reference mixture.

After 24 h of curing, the largest UPV is measured in the concrete specimens made with ash F6. Compared to mixture M0, this increase is 3% in both mixtures F6-5 and F6-15. The accelerated stiffness development in mixture F6-15 continued in the time period from 24–48 h. Šiler et al. [85] attributed fast initial strength development and increased reactivity in mixtures of Portland cement and fluidized bed coal fly ash to the presence of free CaO. The F4 and F6 ashes had free CaO contents of 7.3% and 8.8%, respectively. The differences in the rates of heat evolution and UPV of mixtures containing ash F4 and F6 indicate that this effect had a very small impact on reactivity and stiffness development. 

During the first two days, the mixtures with 15% cement replaced by ash F4 and F5 exhibited slower stiffness development compared to the mixture M0. The results of UPV measurements during the first days of curing correspond well with the results of calorimetry testing. Mixtures containing ash F6 showed an increased rate of heat generation so the accelerated stiffness development can be attributed to an improved hydration process. Mixtures made with ash F4 and F5 exhibited a decrease in heat generation rate so that fewer hydration products were formed within the first days of hydration and the stiffness development is slowed down accordingly.

Measurements over the 2–28-day period indicate that the addition of WFA changed the course of stiffness development. In the reference mixture, the period of 2–7 days shows the largest increase in UPV and a very low increase in UPV from 7–28 days. In mixtures with WFA, the increase in UPV is two to five times larger in the period of 7–28 days compared to the reference mixture. This could be due to the reactivity of ash with the species present in the pore solution.

### 3.5. Apparent Porosity and Capillary Absorption

The average values of apparent porosity ranged from 12.4% to 14.5% (Table 3), which is in agreement with the data obtained in [86] for concrete with a *w/c* ratio of 0.5. Specimens from mixtures containing ashes F4 and F5 with 15% and 30% replacement levels had only slightly increased porosity compared to mixture M0. A significant increase in porosity was only present in specimens with 45% replacement with ash F5. An opposite effect on porosity was observed for specimens with ash F6, where porosity decreased with the increase in cement replacement level. It was found that the incorporation of biomass fly ash increased the pore content of C-S-H compared to the mixtures made with coal fly ash [87]. The decrease in apparent porosity for mixtures with ash F6 was most likely caused by the refinement of pores in cement paste due to improved particle packing. 

**Figure 9:**
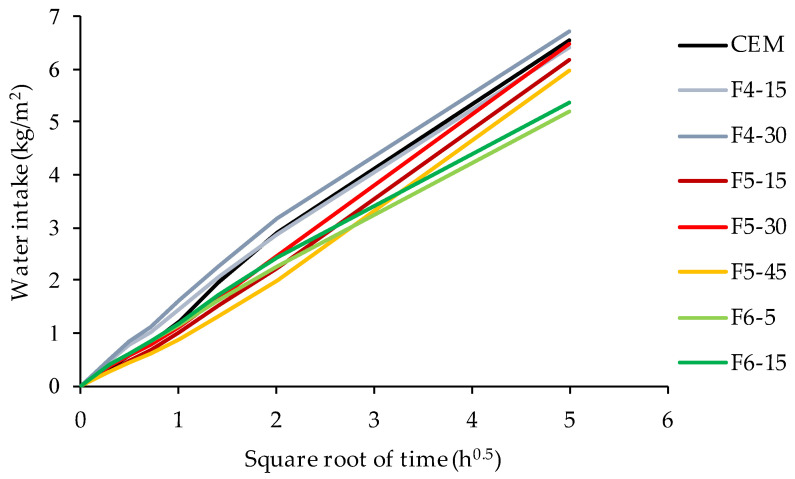
Results of capillary water absorption measurement.

The average values of water intake into the specimens due to capillary absorption are presented in Figure 9. The capillary absorption coefficient for each mixture was calculated and presented in Table 3. The capillary absorption coefficient was calculated using the average slope of the water intake curve over the period 120–1500 min. The initial 120 min of measurement was excluded from regression analysis to reduce the impact of nonlinearities contained in the first period of measurement on the value of the coefficient of capillary absorption. The capillary absorption coefficient and its standard deviation show that the replacement of cement with ash F4 and F5 had no significant influence on capillary absorption, regardless of the amount of cement replacement. Only in mixtures made with ash F6 the capillary absorption coefficient was significantly decreased. Like all fluid transport processes through concrete, the rate of sorption is governed by the pore system [56,59]. Therefore, the reduction in sorption rate can be related to the reduced porosity due to the addition of ash F6.

## 4. Conclusions

The chemical and physical properties of WFA are largely influenced by combustion technology, the type and parts of wood used as fuel, and local operating conditions. The incorporation of WFA in concrete as a partial cement replacement can have a large impact on the fresh and hardened concrete properties. Mixtures made with WFA F6 with a particle size distribution close to that of cement exhibited increased cohesivity, which in turn reduced slump and required more energy for compaction of concrete. At the same time, this type of ash accelerated hydration and stiffness development. The increased cohesiveness of the fresh concrete resulted in concrete with a denser structure and lower porosity, which had a positive influence on the compressive strength and permeability. All these effects can be explained by the filler and filling mechanisms. Mixtures made with WFA F4 that had a similar chemical composition to ash F6 but consisted of coarser particles showed no evidence of the filler effect mechanisms. This suggests that governing mechanisms introduced by the cement replacement with fly ash are connected to the physical interactions between the phases.

When WFA is used as a cement replacement, the workability of the concrete is influenced by the quantity, porosity, and particle size distribution of the WFA. The water demand of the concrete is balanced by the amount of water adsorbed by the WFA particles and the amount of released entrapped water due to the filling effect. The results obtained on mixtures containing F4 and F6 ashes indicate that there may be an optimum content of WFA at which the water demand could be reduced. Increasing the WFA content beyond the optimum will increase the quantity of water required to achieve targeted workability.

The research presented shows that replacing up to 45% of the cement with WFA has very little effect on capillary absorption and can give concrete with a sufficiently high compressive strength to be suitable for construction purposes. Therefore, from the standpoint of thinking of concrete as storage for large quantities of WFA produced, it seems that the construction industry can give a large contribution in this area by using it as a filler material rather than as a cement replacement.

## Figures and Tables

**Table 1:** Chemical and physical composition of cement and WFA [35].

	CEM	F4	F5	F6
Combustion technology		Grate combustor	Grate combustor	Bubbling fluidized bed
Incineration temperature (°C)	-	700–950	up to 800	up to 850
Additive used		-	-	Quartz sand
Type of wood		beech, oak, fir, spruce	beech, oak, hornbeam	beech, oak, hornbeam, poplar
P_2_O_5_	0.22	1.82	1.35	4.03
Na_2_O	0.85	0.65	1.32	0.63
K_2_O	1.25	6.05	4.77	6.21
CaO	59.80	46.75	16.25	47.35
MgO	2.01	8.26	4.30	4.71
Al_2_O_3_	4.94	6.16	10.50	3.56
TiO_2_	0.23	0.34	1.17	0.25
Fe_2_O_3_	3.15	2.85	4.23	1.69
SiO_2_	21.88	19.80	39.95	14.45
SO_3_	3.33	2.73	0.60	3.95
CaCO_3_	6.56	8.13	7.12	26.94
Pozzolanic oxides (SiO_2_ + Al_2_O_3_ + Fe_2_O_3_)	29.97	28.81	54.68	19.70
Alkalies (Na_2_O + 0.658 K_2_O)	1.67	4.63	4.59	4.72
LOI (at 950 °C)	3.60	3.80	8.30	12.70
pH	12.86	13.15	12.97	13.22
*d*_50_ (µm)	9.4	71.9	120.7	17.8
SSA ^1^ (kg/m^2^)	796	223	180	627
Density (g/cm^3^)	3.10	2.59	2.63	2.33
Bulk density (g/cm^3^)	-	0.91	0.61	0.55

^1^ SSA—specific surface area calculated from particle size distribution.

**Table 2:** Concrete mix composition (quantities per 1 m^3^ of concrete).

Mix Designation	M0	F4-15	F4-30	F5-15	F5-30	F5-45	F6-5	F6-15
Cement (kg)	380	323	266	323	266	209	361	323
WFA cement replacement (%)	0	15	30	15	30	45	5	15
WFA content (kg)	0	57	114	57	114	171	19	57
Cement + WFA (kg)	380							
w/(cem. + WFA) ratio	0.5							
Water (kg)	190							
Aggregate (kg)	1821	1811	1801	1811	1801	1791	1816	1805
Fine aggregate (kg)	648	645	641	645	641	638	646	643
Coarse aggregate (kg)	1173	1167	1160	1167	1160	1154	1169	1162

**Table 3:** Properties of fresh and hardened concrete.

Mix Designation	M0	F4-15	F4-30	F5-15	F5-30	F5-45	F6-5	F6-15
Fresh density (kg/m^3^)	2470	2470	2460	2470	2450	2440	2480	2500
Initial temperature (°C)	22.2	23.6	25.1	23.2	23.3	23.3	24.4	22.2
Air content (%)	1.0	0.9	1.1	1.4	0.7	0.3	0.3	1.6
Slump (mm)	90	100	40	85	90	90	110	5
Bulk dry density (kg/m^3^)	2371	2360	2348	2363	2350	2319	2373	2390
	(±8)	(±16)	(±13)	(±13)	(±15)	(±20)	(±24)	(±8)
Bulk saturated density (kg/m^3^)	2506	2499	2484	2500	2485	2464	2503	2514
	(±6)	(±12)	(±10)	(±15)	(±10)	(±12)	(±17)	(±6)
Apparent solid density (kg/m^3^)	2743	2742	2718	2739	2719	2713	2726	2728
	(±4)	(±4)	(±6)	(±22)	(±5)	(±3)	(±8)	(±2)
Apparent porosity (%)	13.57	13.93	13.60	13.73	13.57	14.52	12.94	12.40
	(±0.17)	(±0.48)	(±0.30)	(±0.21)	(±0.49)	(±0.85)	(±0.64)	(±0.27)
Compressive strength (MPa)	45.9 (±0.9)	43.2 (±0.3)	31.9 (±1.4)	45.3 (±0.6)	37.3 (±1.5)	30.1 (±0.8)	48.9 (±1.2)	54.5 (±0.9)
Capillary absorption coefficient (kg/(m^2^h^0.5^))	1.28 (±0.07)	1.22 (±0.11)	1.24 (±0.04)	1.30 (±0.11)	1.35 (±0.13)	1.30 (±0.18)	0.99 (±0.20)	1.02 (±0.07)

Numbers in brackets are standard deviations.

## Data Availability

Not applicable.
